# Understanding the Relationship between Trauma Exposure and Depression among Adolescents after Earthquake: The Roles of Fear and Resilience

**DOI:** 10.3389/fpsyg.2016.02044

**Published:** 2016-12-26

**Authors:** Xiao Zhou, Xinchun Wu, Yuanyuan An

**Affiliations:** ^1^Beijing Key Laboratory of Applied Experimental Psychology, School of Psychology, Beijing Normal UniversityBeijing, China; ^2^The Bob Shapell School of Social Work, Tel Aviv UniversityTel Aviv, Israel; ^3^School of Psychology, Nanjing Normal UniversityNanjing, China

**Keywords:** trauma exposure, fear, resilience, depression, adolescent

## Abstract

Middle school students (*N* = 1435) were assessed 18 months after the Wenchuan earthquake using measures of trauma exposure, fear, resilience, and depression, to examine the effects of fear and resilience on the relationship between trauma exposure and depression. Fear mediated the relationship between trauma exposure and depression, whereas resilience moderated the relationship between fear and depression. These findings suggest that trauma exposure has a direct positive impact on depression, but also indirectly affects depression through fear. Moreover, fear positively predicted depression under conditions of low resilience, whereas this effect was not significant when resilience was high. These results are discussed in terms of their implications for adolescents after trauma.

## Introduction

Many negative psychological reactions can occur after trauma experience (e.g., Armour et al., 2014; Spinhoven et al., 2014), with depression and post-traumatic stress disorders (PTSD) representing two common and critically important negative psychological outcomes (e.g., Lai et al., 2013; Kukihara et al., 2014). Depression and PTSD can co-occur in traumatized individuals (e.g., Elhai et al., 2008; Rytwinski et al., 2013). Nevertheless, a growing number of studies indicate that depression is more prevalent and endurable in traumatized populations with compared with PTSD (e.g., Ying et al., 2013; Cao et al., 2015), and depression may be an important risk factor for PTSD (e.g., Merriman et al., 2007; Ying et al., 2012). The present study therefore focused on depression as a key outcome post-trauma.

Depression refers to a set of negative emotional states (e.g., Klerman, 1977), which have been documented in various populations following different traumatic events (e.g., Otto et al., 2006; Leserman, 2008; Roth et al., 2008). In particular, depression among adolescents after earthquakes has attracted growing interest (e.g., Giannopoulou et al., 2006; Goenjian et al., 2011), because of the observed susceptibility of adolescents to trauma following natural disasters (e.g., Margolin et al., 2010). Prevalence rates for depression have ranged from 13.6 to 51.3% in adolescents exposed to earthquakes (e.g., Kolaitis et al., 2003; Fan et al., 2011; Qu et al., 2012; Ying et al., 2014). The aim of this study was to examine possible predictors and underlying mechanisms for post-earthquake depression.

A predisposing factor for depression following trauma might be the degree of traumatic exposure, according to the work of Freedy et al. (1992), who found that depressive symptoms were related to objective elements of individual trauma experience such as witnessing the disaster, death/injuries of family members, and damage to one’s home (e.g., Goenjian et al., 2009; Ying et al., 2014). In particular, a link had been shown between trauma exposure and depression, such that individuals who experience adverse life events are more than twice as likely to exhibit depression compared with those with no trauma history (e.g., Roberts et al., 2009). Here, the shattered world assumption has been proposed as a possible explanation (Janoff-Bulman, 2010), suggesting that traumatic experiences can challenge people’s stable basic perceptions of personal worth, trust in others, and justice or predictability in the world. This can lead to negative attitudes about self, others, and the world, and in turn result in negative outcomes such as depression.

Additionally, Janoff-Bulman (2010) also emphasized that once these assumptions are severely challenged by traumatic events, they become unstable. Subsequently, trauma survivors can lose a sense of perceived control or predictability in the world. This would lead survivors to experience more fear (Mikkelsen and Einarsen, 2002; Janoff-Bulman, 2010), which refers to a feeling state in which traumatized individuals was afraid to traumatic clues, and worry about some terrible things happening to them again. This state may limit individuals’ cognitive range (e.g., Forbes et al., 2008; Farnsworth and Sewell, 2011) and make it more difficult for people to redirect their attention from negative outcomes and effects of traumatic events. This fixation may increase depression severity. For example, some studies have shown that fear is the most important predictor of depression severity for traumatized individuals (e.g., Başoglu et al., 2004; Ying et al., 2014). It is therefore likely that traumatic exposure may have an indirect effect on depression via fear.

Although effects of traumatic exposure on depression have been identified (e.g., Goenjian et al., 2009; Ying et al., 2014), recent studies have showed that not all individuals exposed to a trauma will go on to develop adverse psychological outcomes (e.g., Lilly et al., 2010; Nygaard and Heir, 2012). For example, Fan et al. (2015) found that 65.3% adolescent survivors may show no adverse psychological outcomes. One study found that 72.5% of adolescent survivors showed no depression after the Wenchuan earthquake (Ye et al., 2014). As such, considerable attention is now paid to individual resilience following trauma (e.g., Bonanno et al., 2011). Here, resilience refers to a constellation of characteristics that enable individuals to adapt to the circumstances they encounter, such as optimism, hardiness, good self-esteem, and social problem solving skills (e.g., Connor and Davidson, 2003).

Resilience and the roles it plays have been investigated in the context of trauma. Such studies have found, for instance, that resilient trauma survivors show characteristics such as hardness, self-enhancement, and optimism (e.g., Bonanno, 2008). These resilient characteristics could work to increase traumatic survivors’ self-esteem and self-enhancement (e.g., Paulhus, 1998), and help survivors develop the belief that one can influence one’s surroundings and the outcome of events and that one can learn and grow from both positive and negative life experiences (e.g., Florian et al., 1995), which in turn may help traumatic survivors cope successfully and find meaningful purpose in life after trauma. Resilient trauma survivors have fewer adverse psychological outcomes such as depression, as compared to less resilient counterparts (e.g., Bonanno, 2008).

A growing body of evidence also suggests that resilience exerts a buffering role in the relationship between traumatic experiences and adverse psychological outcomes after trauma (e.g., Kobasa et al., 1982; Pinquart, 2009). Resilient traumatic survivors have a positive outlook on their surroundings, can make positive re-appraisals of trauma related cues, and considered the trauma as less threatening (e.g., Florian et al., 1995). Resilience may intervene between the experience of a trauma event and survivors’ return to optimism in the face of trauma (e.g., Bonanno et al., 2007), which could work to reduce trauma-related depression (e.g., Andreescu et al., 2007; Sharpley et al., 2014).

Additionally, resilience also can buffer the effect of fear on various post-traumatic outcomes. The work of Block and Kremen (1996) suggested that highly resilient people are characterized by their ability to exert appropriate and dynamic self-regulation, which could help traumatic survivors to regulate themselves in the face of negative emotion (e.g., Waugh et al., 2008), and decrease the effect of fear on negative outcomes after trauma. On the other hand, less resilient people tend to rigidly under or over self-regulate, which could lead to persistence of post-traumatic symptoms (e.g., Waugh et al., 2008).

**FIGURE 1:**
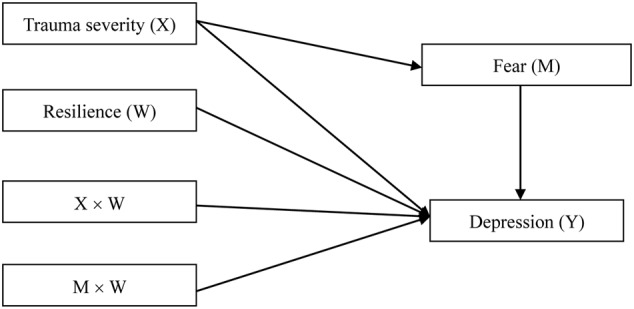
**Proposed moderated mediation**.

Fear could play a mediating role in the relationship between traumatic exposure and depression, whereas resilience could play a moderating role. However, this proposal of direct and indirect effects has yet to be formally evaluated. The present study begins to fill this gap in the literature. Specifically, it was hypothesized that fear would mediate the relationship between traumatic exposure and depression, and that resilience would moderate the relationships between traumatic exposure and depression as well as fear and depression (**Figure 1**).

## Materials and Methods

### Participants

The sample consisted of 1435 adolescent survivors of the Wenchuan earthquake. The mean age of participants at the time of measurement was 14.44 (*SD* = 1.65) years, and the age range was 11.0–19.0 years. Of the 1435 participants, 476 were from senior middle schools and 959 from junior middle schools; 786 were female and 640 were male, and nine did not report gender.

### Procedure

Eighteen months after the earthquake, we focused on Wenchuan and Maoxian counties in Sichuan province, which were most severely affected. We informed local education authorities about the aims and methods of investigation for this study, and indicated that we could provide psychological services if and when they were required. With the help of the local education authorities, we selected middle schools in Wenchuan and Maoxian counties. We then randomly selected several classes with the approval of these schools. All students in selected classrooms were attending school on the assessment date.

This study was approved by the Research Ethics Committee of Beijing Normal University and was conducted with the permission of the principals of the participating schools. Everyone in the selected classes who attended school on the date of the survey was recruited to participate. There were no exclusion criteria. Compensation was not provided. The purpose of the study and the voluntary nature of the students’ participation were highlighted before the survey, and written informed consent was obtained from school principals and classroom teachers. In China, research projects that are approved by local education authorities and the school administrators, and that are deemed to provide a service to the students, do not require parental consent. Assessments were conducted under the supervision of trained individuals with Master’s degrees in psychology. Participants were initially asked to provide demographic information, including sex and age, and then completed measures that assessed traumatic exposure, fear, resilience, and other post-traumatic outcomes. After the questionnaire packets were completed, participants were told that school psychologists or teachers were available to provide psychological/counseling services if needed.

### Measures

#### Trauma Exposure

The trauma exposure questionnaire developed by Wu et al. (2013) was adopted to measure the severity of adolescent survivors’ traumatic experiences. This questionnaire consists of 18 items and asks participants to indicate whether they have directly seen or indirectly heard about the death, injury, or entrapment of parents, friends, teachers, or others. Each of the items is rated on a 3-point scale, where 2 represents “*saw myself,”* 1 represents “*heard about through others,”* and 0 represents *“did not experience the situation above.”* In this study, the internal reliability of the questionnaire was good (α = 0.90).

#### Fear

Fear was measured using the subjective fear questionnaire (Wu et al., 2013), which consists of items assessing fear or worry about the death of parents, friends, teachers, or others. Each of the eight items (e.g., *I fear that my parents will die in the earthquake*) is scored dichotomously with 0 = *no* and 1 = *yes*. In this study, the internal reliability of the questionnaire was again good (α = 0.89).

#### Resilience

Resilience was assessed using the Chinese version (Yu and Zhang, 2007) of the CD-RISC (Connor and Davidson, 2003), a 25-item instrument that assesses the ability to cope with stress and adversity. The items are rated on a 5-point Likert scale ranging from 0 (not true at all) to 4 (true nearly all of the time). Higher scores indicate higher levels of trait resilience. A previous study demonstrated that the scale has good psychometric properties in both the general population and patient samples (Connor and Davidson, 2003). The Chinese version of the CD-RISC was first translated and used by Yu and Zhang (2007) and was found to have good internal consistency, convergent validity, and discriminant validity in adolescent samples (Ying et al., 2016). *Cronbach’s* α of this scale in the present study was 0.94.

#### Depression

Adolescents’ depressive symptoms were measured using the Center for Epidemiologic Studies Depression Scale for Children (Fendrich et al., 1990). The CES-DC is a 20-item self-report measure for the assessment of emotional, cognitive, and behavior-related symptoms of depression. For each item, participants are instructed to assess the frequency of their reactions during the past week. All items are evaluated with 4-point response options (0 = “not at all,” 1 = “a little,” 2 = “some,” 3 = “a lot”). Total possible scores range from 0 to 60, with higher CES-DC scores indicating increased levels of depressive symptoms. The CES-DC has demonstrated good psychometric properties (Barkmann et al., 2008). The Chinese version of the CES-DC has also been found to have good reliability and construct validity among various Chinese populations (e.g., Li et al., 2010; Ying et al., 2013). The *Cronbach’s*α of the scale in the present study was 0.83.

### Data Analysis Strategies

Statistical analyses were conducted using SPSS 17.0. Before statistical analyses, we conducted an analysis of missing data in variables, and found that the missing data across all items totaled less than 3.3% of possible responses. To assess whether the data was missing at random (MAR), we conducted analyses for all variables, using Little’s Missing Completely at Random (MCAR) test. The analysis revealed that the data were indeed MAR, χ^2^ (14) = 18.90, *p* = 0.169. We used lineal imputations to handle cases of missing data.

Descriptive analyses were conducted for all of the measures administered. We firstly considered gender as the categorical variable and examined the gender differences in main variables. Next, *Pearson* correlations were calculated between age, trauma severity, fear, resilience, and depression. We then controlled for gender and age in later moderated mediation analysis by according to the results of gender differences and associations between age and main variables. In analyzing moderated mediation, all independent variables were centered on their respective means to reduce multicollinearity between the main effects and interaction terms, and to increase the interpretability of the interaction term coefficients (e.g., Cohen et al., 2013).

Then, we followed Hayes’s (2013) procedures of moderated mediation analysis of Hayes’s (2013) Statistical Model 15 (**Figure 1**), first examined the moderating effect of resilience on the relationship between trauma severity and depression, and then examined the mediating role of fear in the association between trauma severity and depression. Finally, we assess the moderating effect of resilience on the second stage of the indirect effects (e.g., the relationship between fear and depression). If the effect of the fear on the depression depends on the resilience, then the effect of the trauma severity on the fear should be significant, and that the conditional indirect effect of the trauma severity on the depression via the fear depends on the presence of a certain range of the moderator (e.g., Hayes, 2015). When results above were identified, moderated mediation would be successfully demonstrated.

We conducted bias-corrected bootstrap tests with a 95% confidence interval to test the significance of the indirect effect of trauma severity on depression via fear. Finally, we used the test of simple slopes to further examine the significance of the interaction effects.

## Results

### Descriptive Statistics and Correlations among Main Measures

Descriptive statistics and the correlations among the various measures are shown in **Table 1**. The mean levels of trauma exposure, fear, resilience, and depression were 3.23 (*SD* = 5.03), 5.26 (*SD* = 2.84), 54.24 (*SD* = 19.11), and 20.77 (*SD* = 9.99), respectively. Male students’ mean level of trauma exposure, fear, resilience, and depression were 3.36 (*SD* = 5.44), 4.89 (*SD* = 3.00), 53.51 (*SD* = 20.51), and 19.54 (*SD* = 9.83), respectively. Female students’ mean level of trauma exposure, fear, resilience, and depression were 3.10 (*SD* = 4.66), 5.56 (*SD* = 2.67), 54.91 (*SD* = 17.84), and 21.71 (*SD* = 9.98), respectively. The mean levels of fear and depression among female students were also higher than that among male students [*t* (1424)_Fear_ = -4.37; *t*(1424)_Depression_ = -3.73, *p* < 0.001], but there were no significant gender differences in other variables. In addition, though all of the adolescents experienced the earthquake, they experienced it to different degrees. To be specific, 59.3% (*n* = 798) of them directly saw or indirectly heard about the death, injury, or entrapment of parents, friends, teachers, or others. Furthermore, according to the criterion that 15 is a cutoff indicative of depression (e.g., Weissman et al., 1980), the prevalence of depression was 62.8% (*n* = 845) in the present study.

**Table 1:** Means and standard deviations for and correlations between age, trauma severity, fear, resilience, and depression.

	Mean ±*SD*	1	2	3	4	5
(1) Age	14.44 ± 1.65	–	0.03	0.02	0.13^∗∗^	0.14^∗∗^
(2) Trauma exposure	3.23 ± 5.03	0.03	–	0.17^∗∗^	0.07^∗∗^	0.18^∗∗^
(3) Fear	5.26 ± 2.84	0.02	0.17^∗∗^	–	0.14^∗∗^	0.15^∗∗^
(4) Resilience	54.24 ± 19.11	0.13^∗∗^	0.07^∗∗^	0.14^∗∗^	–	-0.09^∗∗^
(5) Depression	20.77 ± 9.99	0.13^∗∗^	0.18^∗∗^	0.15^∗∗^	-0.08^∗∗^	–

*^∗∗∗^*p* < 0.001, ^∗∗^*p* < 0.01, ^∗^*p* < 0.05. Correlation coefficients before omitting one depression item were in the bottom-left boxes, and that after omitting were in the upper-right boxes.*

Next, *Pearson* correlations among the main variables were calculated. These analyses found that age was significantly related to resilience and depression. Additionally, trauma exposure was positively related to fear, resilience, and depression, fear was positively related to resilience, and resilience was negatively associated with depression. Considering the potential overlap between the content of fear and depression, we re-examined the correlations between main variables after omitting one item of depression (e.g., I am scared). The results showed no essential change (**Table 1**).

### Moderated Mediation Analysis

Based on the descriptive statistics and correlation results reported above, we controlled for age and gender in following five regression equations. Regression equations 1 and 2 were constructed to examine the effects of trauma exposure on depression and fear. We found that trauma exposure had significant positive effects on both depression and fear. Next, we examined whether resilience moderates the relationship between trauma exposure and depression (equation 3). We found that resilience directly predicted depression, whereas the interaction between trauma exposure and resilience was not significant, indicating that resilience does not moderate the relationship between trauma exposure and depression. In equation 4, fear and trauma exposure significantly predicted depression even in a combined model. Given the results for equations 2 and 4, we can conclude that fear mediates the relationship between trauma exposure and depression. In equation 5, the moderating effect of resilience on the association between fear and depression was evaluated. We found that the interaction between fear and resilience was significant and negative. This finding indicates that resilience does moderate the relationship between fear and depression. In conclusion, our results suggest that fear mediates the relationship between trauma exposure and depression and that resilience moderates the relationship between fear and depression.

In addition, considering the potential overlap between the content of fear and depression, we re-did a moderated mediation analysis following the above procedures after omitting one item of depression. The results also showed no essential change compared with the original results (**Table 2**). Thus, we did not re-do a second bias-corrected bootstrap test and simple slopes analysis anymore.

**Table 2:** Regression analysis results: Testing fear as a mediator and resilience as a moderator in the relation between trauma exposure and depression.

	Equation 1		Equation 2		Equation 3		Equation 4		Equation 5	
	Variable (depression)		Variable (fear)		Variable (depression)		Variable (depression)		Variable (depression)	
	β	*t*	β	*t*	β	*t*	β	*t*	β	*t*
Gender	0.09 (0.08)	2.94^∗∗^ (2.67^∗∗^)	0.11 (0.11)	3.89^∗∗∗^ (3.89^∗∗∗^)	0.09 (0.08)	2.95^∗∗^ (2.70^∗∗^)	0.07 (0.07)	2.50^∗^ (2.27^∗^)	0.08 (0.07)	2.78^∗^ (2.38^∗^)
Age	0.11 (0.12)	3.84^∗∗∗^ (4.02^∗∗∗^)	0.002 (0.002)	-0.02 (-0.02)	0.14 (0.14)	4.56^∗∗∗^ (4.76^∗∗∗^)	0.14 (0.14)	4.62^∗∗∗^ (4.83^∗∗∗^)	0.13 (0.14)	4.52^∗∗∗^ (4.73^∗∗∗^)
Trauma exposure	0.18 (0.18)	6.14^∗∗∗^ (6.08^∗∗∗^)	0.18 (0.18)	6.05^∗∗∗^ (6.05^∗∗∗^)	0.20 (0.19)	6.57^∗∗∗^ (6.53^∗∗∗^)	0.17 (0.17)	5.83^∗∗∗^ (5.78^∗∗∗^)	0.18 (0.17)	5.90^∗∗∗^ (5.85^∗∗∗^)
Resilience					-0.16 (-0.16)	-5.62^∗∗∗^ (-5.42^∗∗∗^)	-0.17 (-0.17)	-5.58^∗∗∗^ (-5.73^∗∗∗^)	-0.17 (-0.18)	-5.80^∗∗∗^ (-5.94^∗∗∗^)
Trauma exposure × Resilience					0.001 (0.001)	0.03 (0.03)				
Fear							0.12 (0.12)	4.00^∗∗∗^ (3.93^∗∗∗^)	0.11 (0.11)	3.68^∗∗∗^ (3.65^∗∗∗^)
Fear × Resilience									-0.09 (-0.09)	-3.00^∗∗^ (-3.09^∗∗^)
*R*^2^	0.06 (0.06)		0.04 (0.04)		0.08 (0.08)		0.09 (0.09)		0.10 (0.10)	
*F*	21.54^∗∗∗^ (21.19^∗∗∗^)		18.88^∗∗∗^ (18.88^∗∗∗^)		18.74^∗∗∗^ (18.89^∗∗∗^)		21.67^∗∗∗^ (21.77^∗∗∗^)		19.70^∗∗∗^ (19.88^∗∗∗^)	

*^∗∗∗^*p* < 0.001, ^∗∗^*p* < 0.01, ^∗^*p* < 0.05. The regression coefficients before omitting one depression item were outside brackets, and that after omitting were inside brackets.*

**FIGURE 2:**
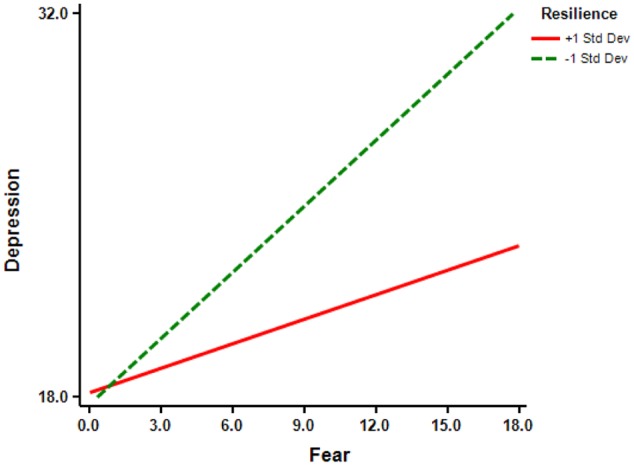
**Relationship between fear and depression at different levels of resilience**.

To further test the significance of the mediation effect, we conducted a bias-corrected bootstrap test with a 95% confidence interval. The results revealed a 95% confidence interval from 0.02 to 0.07, indicating that fear does mediate the relationship between trauma exposure and depression, according to Preacher and Hayes (2008) guidelines. Similarly, we used the test of simple slopes to further examine whether the moderating effect of resilience was significant. We graphed the relationship between fear and depression for participants whose resilience levels were 1 SD above or below the mean (**Figure 2**).

For participants 1 SD below the mean for resilience, increased fear was associated with a significant increase in depression (*simple slope* = 0.80, *t* = 6.02, *p* < 0.001). In contrast, for participants 1 SD above the mean for resilience, increased fear was not significantly associated with a change in depression (*simple slope* = 0.30, *t* = 1.91, *p* > 0.05).

## Discussion

The present cross-sectional study sought to examine the role of fear and resilience in the association between trauma exposure and depression. Firstly, we assessed the prevalence of depression in this study, and found that the prevalence was 62.8% among adolescents 18 months after the Wenchuan earthquake, higher than that (e.g., 27.6, 40.6, 30.9, 37.5, and 29.8% after 6, 12, 18, 24, and 30 months since earthquake) found in the studies from Fan’s team (e.g., Shi et al., 2016; Zhou et al., 2016). The difference can be attributed to the different participants. Specifically, the adolescents Fan’s team investigated were from Dujiangyan city, about 20 kms away from the epicenter (e.g., Shi et al., 2016; Zhou et al., 2016). However, the Wenchuan and Maoxian county, where the adolescents in the current study came from, are the epicenter. Wherein, the adolescents might have severer trauma exposure, and hence experience higher prevalence of depression. In addition, compared with our previous studies, the prevalence of depression 18 months after the Wenchuan earthquake in this study was higher than that 1 year after earthquake (e.g., 42.5%; Ying et al., 2013), but lower than that 30 months after earthquake (e.g., 69.5%; Lin et al., 2013). That is, the findings further supported our prior conclusion that the depression of adolescents in Wenchuan and Maoxian county after the Wenchuan earthquake had an increasing trajectory over 30 months (e.g., Wu et al., 2015).

Next, by using series of regression equation, we examined the mediating role of fear and the moderating role of resilience in the association between trauma exposure and depression. Firstly, we found that trauma exposure was positively associated with depression. This is consistent with previous studies (e.g., Goenjian et al., 2009; Ye et al., 2014; Ying et al., 2014) and supports the shattered world assumption (Janoff-Bulman, 2010), which indicates that traumatic experience is a prerequisite for post-traumatic depression.

Furthermore, this study also found that fear mediated the relationship between traumatic exposure and depression, which indicates that traumatic exposure has a positive and indirect association with depression via fear. We posit that trauma causes adolescent survivors to lose their sense of control in the world, producing fear (e.g., Foa et al., 1995), which may in turn increase mental stress (e.g., Nolen-Hoeksema, 1991), and lead to negative outcomes such as depression. Additionally, fear of traumatic clues also could lead to conditioned fear reactions (e.g., Jovanovic et al., 2009) and elicit general worry about anything related to such traumatic clues, which could in turn result in depression. Moreover, adolescents after earthquake were exposed to the threatening surrounding that would elicit their fear reaction. Fear then limited adolescents’ cognitive range (e.g., Farnsworth and Sewell, 2011), making it difficult for them to distract attention from the negative outcomes following earthquake. Ultimately, adolescents’ negative cognition increased and then depression would elevate (e.g., Ciesla and Roberts, 2007; Oei and Kwon, 2007).

In addition, we found that resilience was negatively associated with depression, likely acting as a protective factor. This is consistent with previous studies that emphasize the adaptive function of resilience in trauma contexts (e.g., Wingo et al., 2010; Ying et al., 2014). One possible reason is that resilience connotes strength, flexibility, a capacity for mastery, and resumption of normal functioning after trauma (e.g., Richardson, 2002), and reflects a pattern of competence and self-efficacy (e.g., Agaibi and Wilson, 2005). These factors could help adolescences to cope positively with negative outcomes due to trauma (e.g., Caffo and Belaise, 2003).

Nevertheless, we also found a positive relation between trauma exposure and resilience in correlation analysis. This is consistent with Bensimon’s (2012) finding on the relation between traumatic events and resilience, and parallels with Collins’ (2009) results that showing the positive relation between life events and resilience. Traumatic event can elicit individuals’ cognitive disequilibrium by challenging their stable cognitive system, and in such way their cognitive process or reconfiguration will be activated (e.g., Janoff-Bulman, 2010). During the process of reconstruction, traumatized individuals will experience more resilience (e.g., Walsh, 2007; Bensimon, 2012). In addition, fear was also found to be positively associated with resilience in correlation analysis. It is well suggested that individuals with high level of resilience have greater sense of mastery (e.g., Richardson, 2002), however, when exposed to a massive traumatic event, their sense of mastery may encounter more serious challenge, which will lead to a severer loss of actual control on the post-traumatic world and thus they experience more fear. As Charney (2004) suggested, resilient people are not fearless but are willing and able to approach a fear-inducing situation despite the presence of subjective fear.

Inconsistent with previous studies (e.g., Campbell-Sills et al., 2006; Wingo et al., 2010), we did not find a moderating role of resilience in the relationship between traumatic exposure and depression. This inconsistency could be attributed to participant differences. The participants of previous studies were adults (e.g., Campbell-Sills et al., 2006; Wingo et al., 2010), but the participants of this study were adolescents who have relatively fewer cognitive capacities compared with adults. This reduced capacity could limit adolescents in terms of understanding the meaning of trauma (e.g., Dai et al., 2014), thereby diminishing the protective effects of resilience, even though individuals with higher resilience may be more likely to consider the trauma in a positive light (e.g., Wilson, 1995).

Consistent with our hypotheses and the study of Ying et al. (2014), our findings indicate that resilience moderates the relationship between fear and depression, and further suggest that resilience buffers the effect of fear on depression. Based on the work of Block and Kremen (1996), we posit that resilient adolescents have effective emotion regulation skills that serve to reduce the effect of fear on depression. Additionally, resilient individuals may be more optimistic compared to individuals with less resilience (e.g., Catalano et al., 2011), such that the effect of fear on depression may be reduced. In addition, traumatized people with high resilience tend to develop a closer bond with a group, to place a higher value on altruism, and to have a greater capacity to tolerate fear and to perform in the same efficiency (e.g., Bell, 2001). Therefore, resilient people are more willing and able to approach a fear-inducing situation despite the presence of subjective fear, and can function effectively at the same time (e.g., Charney, 2004), thus buffering the effect of fear on depression.

Several design and measurement limitations must be acknowledged. First, due to the attrition of participants between measurements, the sample may be somewhat selective. Second, although the correlations and regression coefficients were statistically significant and consistent with our hypotheses, the effect sizes were not particularly large. Moreover, except for gender and age, this study did not take other socio-demographic characteristics (e.g., socioeconomic status, parental education, etc.) into consideration. In addition, this study’s cross-sectional design means that our findings do not indicate causality or a temporal sequence.

Notwithstanding these limitations, the current study is of importance first and foremost because, to the best of our knowledge, it is of the first to examine factors affecting adolescents’ depression after disaster from the perspective of fear and resilience. The findings further support shattered world assumption theory, and indicate that trauma severity might be the primary risk factor, with fear-related processing leading to depression after trauma. Additionally, the results also indicated that resilience has an adaptive function for traumatized adolescents. Taken together, these findings contribute to extant knowledge concerning the relationship between traumatic exposure and depression.

From a clinical perspective, our results suggest that clinical efforts should focus on decreasing fear. Then, repeatedly exposing a client for prolonged periods to a feared object or traumatic clues in the company of a supportive therapist (e.g., Davis et al., 2006), which can make adolescents habituate to these cues, and thus reduce the fear response (e.g., Hofmann, 2008), which in turn can lead to less depression. Additionally, it is also important for school psychologists to promote resilience, and to encourage the development of factors associated with greater resilience in high-risk children (e.g., Velleman and Templeton, 2007). For example, teachers can provide adolescents with more supports and help them to improve self-esteem and self-efficacy, and these in turn can lead to more resilience (e.g., Dumont and Provost, 1999; Veselska et al., 2009).

## Author Contributions

XZ contribute to write the overall manuscripts. XW and YA contribute to revise the manuscript.

## Conflict of Interest Statement

The authors declare that the research was conducted in the absence of any commercial or financial relationships that could be construed as a potential conflict of interest.

The reviewer DV and the handling Editor declared their shared affiliation, and the handling Editor states that the process nevertheless met the standards of a fair and objective review.

## References

[B1] Agaibi C. E. Wilson J. P. (2005). Trauma, PTSD, and resilience: a review of the literature. *Trauma Violence Abuse* 6 195–216. 10.1177/1524838005277438 16237155

[B2] Andreescu C. Lenze E. J. Dew M. A. Begley A. E. Mulsant B. H. Dombrovski A. Y. (2007). Effect of comorbid anxiety on treatment response and relapse risk in late-life depression: controlled study. *Br. J. Psychiatry* 190 344–349. 10.1192/bjp.bp.106.027169 17401042

[B3] Armour C. Elklit A. Lauterbach D. Elhai J. D. (2014). The DSM-5 dissociative-PTSD subtype: can levels of depression, anxiety, hostility, and sleeping difficulties differentiate between dissociative-PTSD and PTSD in rape and sexual assault victims? *J. Anxiety Disord.* 28 418–426. 10.1016/j.janxdis.2013.12.008 24568742

[B4] Barkmann C. Erhart M. Schulte-Markwort M. Group B. S. (2008). The German version of the centre for epidemiological studies depression scale for children: psychometric evaluation in a population-based survey of 7 to 17 years old children and adolescents–results of the BELLA study. *Eur. Child Adolesc. Psychiatry* 17 116–124. 10.1007/s00787-008-1013-0 19132311

[B5] Başoglu M. Kiliç C. Şalcioglu E. Livanou M. (2004). Prevalence of posttraumatic stress disorder and comorbid depression in earthquake survivors in Turkey: an epidemiological study. *J. Trauma Stress* 17 133–141. 10.1023/B:JOTS.0000022619.31615.e8 15141786

[B6] Bell C. C. (2001). Cultivating resiliency in youth. *J. Adolesc. Health* 29 375–381. 10.1016/S1054-139X(01)00306-8 11691598

[B7] Bensimon M. (2012). Elaboration on the association between trauma, PTSD and posttraumatic growth: the role of trait resilience. *Pers. Individ. Dif.* 52 782–787. 10.1016/j.paid.2012.01.011

[B8] Block J. Kremen A. M. (1996). IQ and ego-resiliency: conceptual and empirical connections and separateness. *J. Pers. Soc. Psychol.* 70 349–361. 10.1037/0022-3514.70.2.349 8636887

[B9] Bonanno G. A. (2008). Loss, trauma, and human resilience: Have we underestimated the human capacity to thrive after extremely aversive events? *Psychol. Trauma* 1 101–113. 10.1037/1942-9681.S.1.101 14736317

[B10] Bonanno G. A. Galea S. Bucciarelli A. Vlahov D. (2007). What predicts psychological resilience after disaster? The role of demographics, resources, and life stress. *J. Consult. Clin. Psychol.* 75 671–682. 10.1037/0022-006X.75.5.671 17907849

[B11] Bonanno G. A. Westphal M. Mancini A. D. (2011). Resilience to loss and potential trauma. *Annu. Rev. Clin. Psychol.* 7 511–535. 10.1146/annurev-clinpsy-032210-104526 21091190

[B12] Caffo E. Belaise C. (2003). Psychological aspects of traumatic injury in children and adolescents. *Child. Adolesc. Psychiatr. N. Am.* 12 493–535. 10.1016/S1056-4993(03)00004-X 12910820

[B13] Campbell-Sills L. Cohan S. L. Stein M. B. (2006). Relationship of resilience to personality, coping, and psychiatric symptoms in young adults. *Behav. Res. Ther.* 44 585–599. 10.1016/j.brat.2005.05.001 15998508

[B14] Cao X. Wang L. Cao C. Zhang J. Liu P. Zhang B. (2015). Patterns of DSM-5 posttraumatic stress disorder and depression symptoms in an epidemiological sample of Chinese earthquake survivors: a latent profile analysis. *J. Affect. Disord.* 186 58–65. 10.1016/j.jad.2015.06.058 26231442

[B15] Catalano D. Chan F. Wilson L. Chiu C.-Y. Muller V. R. (2011). The buffering effect of resilience on depression among individuals with spinal cord injury: a structural equation model. *Rehabil. Psychol.* 56 200–211. 10.1037/a0024571 21843016

[B16] Charney D. S. (2004). Psychobiological mechanisms of resilience and vulnerability: implications for successful adaptation to extreme stress. *Am. J. Psychiatry* 161 195–216. 10.1176/appi.ajp.161.2.195 14754765

[B17] Ciesla J. A. Roberts J. E. (2007). Rumination, negative cognition, and their interactive effects on depressed mood. *Emotion* 7 555–565. 10.1037/1528-3542.7.3.555 17683212

[B18] Cohen J. Cohen P. West S. G. Aiken L. S. (2013). *Applied Multiple Regression/Correlation Analysis for the Behavioral Sciences* 3 Edn. Mahwah, NJ: Lawrence Eribaum Associates.

[B19] Collins A. B. (2009). *Life Experiences and Resilience in College Students: A Relationship Influenced by Hope and Mindfulness.* Doctoral dissertation, Texas A&M University College Station, TX.

[B20] Connor K. M. Davidson J. R. T. (2003). Development of a new resilience scale: the Connor-Davidson resilience scale (CD-RISC). *Depress. Anxiety* 18 76–82. 10.1002/da.10113 12964174

[B21] Dai Y. Lei M. Zhou X. Yao M. Jiang J. Chen X. (2014). The effect of trauma expose on posttraumatic stress disorder after the Wenchuan earthquake: the role of resilience as a moderator[Chinese]. *Psychol. Dev. Educ.* 30 61–67.

[B22] Davis M. Ressler K. Rothbaum B. O. Richardson R. (2006). Effects of D-cycloserine on extinction: translation from preclinical to clinical work. *Biol. Psychiatry* 60 369–375. 10.1016/j.biopsych.2006.03.084 16919524

[B23] Dumont M. Provost M. A. (1999). Resilience in adolescents: protective role of social support, coping strategies, self-esteem, and social activities on experience of stress and depression. *J. Youth Adolesc.* 28 343–363. 10.1023/A:1021637011732

[B24] Elhai J. D. Grubaugh A. L. Kashdan T. B. Frueh B. C. (2008). Empirical examination of a proposed refinement to DSM-IV posttraumatic stress disorder symptom criteria using the National Comorbidity Survey Replication data. *J. Clin. Psychiatry* 69 597–602. 10.4088/JCP.v69n0411 18294026

[B25] Fan F. Long K. Zhou Y. Zheng Y. Liu X. (2015). Longitudinal trajectories of post-traumatic stress disorder symptoms among adolescents after the Wenchuan earthquake in China. *Psychol. Med.* 45 2885–2896. 10.1017/S0033291715000884 25990926

[B26] Fan F. Zhang Y. Yang Y. Mo L. Liu X. (2011). Symptoms of posttraumatic stress disorder, depression, and anxiety among adolescents following the 2008 Wenchuan earthquake in China. *J. Trauma Stress* 24 44–53. 10.1002/jts.20599 21351164

[B27] Farnsworth J. K. Sewell K. W. (2011). Fear of emotion as a moderator between PTSD and firefighter social interactions. *J. Trauma Stress* 24 444–450. 10.1002/jts.20657 21780188

[B28] Fendrich M. Weissman M. M. Warner V. (1990). Screening for depressive disorder in children and adolescents: validating the center for epidemiologic studies depression scale for children. *Am. J. Epidemiol.* 131 538–551. 2301363 10.1093/oxfordjournals.aje.a115529

[B29] Florian V. Mikulincer M. Taubman O. (1995). Does hardiness contribute to mental health during a stressful real-life situation? The roles of appraisal and coping. *J. Pers. Soc. Psychol.* 68 687–695. 10.1037/0022-3514.68.4.687 7738771

[B30] Foa E. B. Riggs D. S. Massie E. D. Yarczower M. (1995). The impact of fear activation and anger on the efficacy of exposure treatment for posttraumatic stress disorder. *Behav. Ther.* 26 487–499. 10.1016/S0005-7894(05)80096-6

[B31] Forbes D. Parslow R. Creamer M. Allen N. McHugh T. Hopwood M. (2008). Mechanisms of anger and treatment outcome in combat veterans with posttraumatic stress disorder. *J. Trauma Stress* 21 142–149. 10.1002/jts.20315 18404639

[B32] Freedy J. R. Resnick H. S. Kilpatrick D. G. (1992). “Conceptual framework for evaluating disaster impact: implications for clinical intervention,” in *Responding to Disaster: A Guide for Mental Health Professionals* ed. Austin L. S. (Washington, DC: American Psychiatric Press) 6–14.

[B33] Giannopoulou I. Strouthos M. Smith P. Dikaiakou A. Galanopoulou V. Yule W. (2006). Post-traumatic stress reactions of children and adolescents exposed to the Athens 1999 earthquake. *Eur. Psychiatry* 21 160–166. 10.1016/j.eurpsy.2005.09.005 16529912

[B34] Goenjian A. K. Roussos A. Steinberg A. M. Sotiropoulou C. Walling D. Kakaki M. (2011). Longitudinal study of PTSD, depression, and quality of life among adolescents after the Parnitha earthquake. *J. Affect. Disord.* 133 509–515. 10.1016/j.jad.2011.04.053 21641650

[B35] Goenjian A. K. Walling D. Steinberg A. M. Roussos A. Goenjian H. A. Pynoos R. S. (2009). Depression and PTSD symptoms among bereaved adolescents 6½12 years after the 1988 spitak earthquake. *J. Affect. Disord.* 112 81–84. 10.1016/j.jad.2008.04.006 18547646

[B36] Hayes A. F. (2013). *Introduction to Mediation, Moderation, and Conditional Process Analysis: A Regression-Based Approach.* New York, NY: Guilford Press.

[B37] Hayes A. F. (2015). An index and test of linear moderated mediation. *Multivariate Behav. Res.* 50 1–22. 10.1080/00273171.2014.962683 26609740

[B38] Hofmann S. G. (2008). Cognitive processes during fear acquisition and extinction in animals and humans: implications for exposure therapy of anxiety disorders. *Clin. Psychol. Rev.* 28 199–210. 10.1016/j.cpr.2007.04.009 17532105 PMC2268629

[B39] Janoff-Bulman R. (2010). *Shattered Assumptions.* New York, NY: Simon and Schuster.

[B40] Jovanovic T. Norrholm S. D. Fennell J. E. Keyes M. Fiallos A. M. Myers K. M. (2009). Posttraumatic stress disorder may be associated with impaired fear inhibition: relation to symptom severity. *Psychiatry Res.* 167 151–160. 10.1016/j.psychres.2007.12.014 19345420 PMC2713500

[B41] Klerman G. L. (1977). “Anxiety and depression,” in *Handbook of Studies on Depression* ed. Burrows G. D. (New York, NY: Excerpta Medica Amsterdam) 49–68.

[B42] Kobasa S. C. Maddi S. R. Kahn S. (1982). Hardiness and health: a prospective study. *J. Pers. Soc. Psychol.* 42 168–177. 10.1037/0022-3514.42.1.168 7057354

[B43] Kolaitis G. Kotsopoulos J. Tsiantis J. Haritaki S. Rigizou F. Zacharaki L. (2003). Posttraumatic stress reactions among children following the Athens earthquake of September 1999. *Eur. Child Adolesc. Psychiatry* 12 273–280. 10.1007/s00787-003-0339-x 14689259

[B44] Kukihara H. Yamawaki N. Uchiyama K. Arai S. Horikawa E. (2014). Trauma, depression, and resilience of earthquake/tsunami/nuclear disaster survivors of Hirono, Fukushima, Japan. *Psychiatry Clin. Neurosci.* 68 524–533. 10.1111/pcn.12159 24444298

[B45] Lai B. S. La Greca A. M. Auslander B. A. Short M. B. (2013). Children’s symptoms of posttraumatic stress and depression after a natural disaster: comorbidity and risk factors. *J. Affect. Disord.* 146 71–78. 10.1016/j.jad.2012.08.041 22974469 PMC3640419

[B46] Leserman J. (2008). Role of depression, stress, and trauma in HIV disease progression. *Psychosom. Med.* 70 539–545. 10.1097/PSY.0b013e3181777a5f 18519880

[B47] Li W. Cheung H. Chung O. K. J. Ho K. Y. (2010). Center for epidemiologic studies depression scale for children: psychometric testing of the chinese version. *J. Adv. Nurs.* 66 2582–2591. 10.1111/j.1365-2648.2010.05440.x 20825514

[B48] Lilly M. M. Valdez C. E. Graham-Bermann S. A. (2010). The mediating effect of world assumptions on the relationship between trauma exposure and depression. *J. Interpers. Violence* 26 2499–2516. 10.1177/0886260510383033 20829232

[B49] Lin C. Wu X. Zhang Y. Zang W. Zhou X. Dai Y. (2013). Investigation on mental health of primary and secondary school students after 30 months of Wenchuan earthquake[Chinese]. *Psychol. Dev. Educ.* 29 631–640.

[B50] Margolin G. Ramos M. C. Guran E. L. (2010). Earthquakes and children: the role of psychologists with families and communities. *Prof. Psychol. Res. Pr.* 41 1–9. 10.1037/a0018103 20428504 PMC2859846

[B51] Merriman C. Norman P. Barton J. (2007). Psychological correlates of PTSD symptoms following stroke. *Psychol. Health Med.* 12 592–602. 10.1080/13548500601162747 17828679

[B52] Mikkelsen E. G. E. Einarsen S. (2002). Basic assumptions and symptoms of post-traumatic stress among victims of bullying at work. *Eur. J. Work Organ. Psychol.* 11 87–111. 10.1080/13594320143000861

[B53] Nolen-Hoeksema S. (1991). Responses to depression and their effects on the duration of depressive episodes. *J. Abnorm. Psychol.* 100 569–582. 10.1037/0021-843X.100.4.569 1757671

[B54] Nygaard E. Heir T. (2012). World assumptions, posttraumatic stress and quality of life after a natural disaster: a longitudinal study. *Health Qual. Life Outcomes* 10 76–83. 10.1186/1477-7525-10-76 22742447 PMC3478202

[B55] Oei T. P. S. Kwon S. M. (2007). Evaluation of the integrated cognitive model of depression and its specificity in a migrant population. *Depress. Anxiety* 24 112–123. 10.1002/da.20225 16888757

[B56] Otto K. Boos A. Dalbert C. Schöps D. Hoyer J. (2006). Posttraumatic symptoms, depression, and anxiety of flood victims: the impact of the belief in a just world. *Pers. Individ. Dif.* 40 1075–1084. 10.1016/j.paid.2005.11.010

[B57] Paulhus D. L. (1998). Interpersonal and intrapsychic adaptiveness of trait self-enhancement: a mixed blessing? *J. Pers. Soc. Psychol.* 74 1197–1208. 10.1037/0022-3514.74.5.1197 9599439

[B58] Pinquart M. (2009). Moderating effects of dispositional resilience on associations between hassles and psychological distress. *J. Appl. Dev. Psychol.* 30 53–60. 10.1016/j.appdev.2008.10.005

[B59] Preacher K. J. Hayes A. F. (2008). Asymptotic and resampling strategies for assessing and comparing indirect effects in multiple mediator models. *Behav. Res. Methods* 40 879–891. 10.3758/BRM.40.3.879 18697684

[B60] Qu Z. Wang X. Tian D. Zhao Y. Zhang Q. He H. (2012). Posttraumatic stress disorder and depression among new mothers at 8 months later of the 2008 Sichuan earthquake in China. *Arch. Women Ment. Health* 15 49–55. 10.1007/s00737-011-0255-x 22249399

[B61] Richardson G. E. (2002). The metatheory of resilience and resiliency. *J. Clin. Psychol.* 58 307–321. 10.1002/jclp.10020 11836712

[B62] Roberts B. Damundu E. Y. Lomoro O. Sondorp E. (2009). Post-conflict mental health needs: a cross-sectional survey of trauma, depression and associated factors in Juba, *Southern Sudan*. *BMC Psychiatry* 9:7 10.1186/1471-244X-9-7 PMC2656503 19261192

[B63] Roth R. S. Geisser M. E. Bates R. (2008). The relation of post-traumatic stress symptoms to depression and pain in patients with accident-related chronic pain. *J. Pain* 9 588–596. 10.1016/j.jpain.2008.01.333 18343728

[B64] Rytwinski N. K. Scur M. D. Feeny N. C. Youngstrom E. A. (2013). The co-occurrence of major depressive disorder among individuals with posttraumatic stress disorder: a meta-analysis. *J. Trauma Stress* 26 299–309. 10.1002/jts.21814 23696449

[B65] Sharpley C. F. Bitsika V. Wootten A. C. Christie D. R. H. (2014). Does resilience ‘buffer’against depression in prostate cancer patients? A multi-site replication study. *Eur. J. Cancer Care* 23 545–552. 10.1111/ecc.12170 24506500

[B66] Shi X. Nancy X. Zhou Y. Geng F. Fan F. (2016). Depressive symptoms and associated psychosocial factors among adolescent survivors 30 months after 2008 Wenchuan earthquake: a follow-up study. *Front. Psychol.* 7:467 10.3389/fpsyg.2016.00467 PMC4811865 27065931

[B67] Spinhoven P. Penninx B. W. van Hemert A. M. de Rooij M. Elzinga B. M. (2014). Comorbidity of PTSD in anxiety and depressive disorders: prevalence and shared risk factors. *Child Abuse Negl.* 38 1320–1330. 10.1016/j.chiabu.2014.01.017 24629482

[B68] Velleman R. Templeton L. (2007). Understanding and modifying the impact of parents’ substance misuse on children. *Adv. Psychiatry Treat.* 13 79–89. 10.1192/apt.bp.106.002386

[B69] Veselska Z. Geckova A. M. Orosova O. Gajdosova B. van Dijk J. P. Reijneveld S. A. (2009). Self-esteem and resilience: the connection with risky behavior among adolescents. *Addict. Behav.* 34 287–291. 10.1016/j.addbeh.2008.11.005 19056183

[B70] Walsh F. (2007). Traumatic loss and major disasters: strengthening family and community resilience. *Fam. Process* 46 207–227. 10.1111/j.1545-5300.2007.00205.x 17593886

[B71] Waugh C. E. Fredrickson B. L. Taylor S. F. (2008). Adapting to life’s slings and arrows: individual differences in resilience when recovering from an anticipated threat. *J. Res. Pers.* 42 1031–1046. 10.1016/j.jrp.2008.02.005 19649310 PMC2711547

[B72] Weissman M. M. Orvaschel H. Padian N. (1980). Children’s symptom and social functioning self-report scales comparison of mothers’ and children’s reports. *J. Nerv. Ment. Dis.* 168 736–740. 10.1097/00005053-198012000-00005 7452212

[B73] Wilson J. P. (1995). “Traumatic events and PTSD prevention,” in *Handbook of Preventative Psychiatry* eds Raphael B. Burrows G. (Amsterdam: Elsevier North-Holland) 281–296.

[B74] Wingo A. P. Wrenn G. Pelletier T. Gutman A. R. Bradley B. Ressler K. J. (2010). Moderating effects of resilience on depression in individuals with a history of childhood abuse or trauma exposure. *J. Affect. Disord.* 126 411–414. 10.1016/j.jad.2010.04.009 20488545 PMC3606050

[B75] Wu X. Zhang Y. Lin C. Zang W. (2013). The effect of disaster exposure on PTSD of primary and secondary students: mediating and moderating effects[Chinese]. *Psychol. Dev. Educ.* 29 641–648.

[B76] Wu X. Zhou X. Lin C. Chen J. (2015). Adolescents’ psychological reactions following traumatic events: influencing mechanism and intervention[Chinese]. *Psychol. Dev. Educ.* 31 117–127.

[B77] Ye Y. Fan F. Li L. Han Q. (2014). Trajectory and predictors of depressive symptoms among adolescent survivors following the Wenchuan earthquake in China: a cohort study. *Soc. Psychiatry Psychiatr. Epidemiol.* 49 943–952. 10.1007/s00127-014-0821-4 24429727

[B78] Ying L. Wang Y. Lin C. Chen C. (2016). Trait resilience moderated the relationships between PTG and adolescent academic burnout in a post-disaster context. *Pers. Individ. Dif.* 90 108–112. 10.1016/j.paid.2015.10.048

[B79] Ying L. Wu X. Lin C. (2012). Longitudinal linkages between depressive and posttraumatic stress symptoms in adolescent survivors following the Wenchuan earthquake in China: a three-wave, cross-lagged study. *School Psychol. Int.* 33 416–432. 10.1177/0143034311421271

[B80] Ying L. Wu X. Lin C. Chen C. (2013). Prevalence and predictors of posttraumatic stress disorder and depressive symptoms among child survivors 1 year following the Wenchuan earthquake in China. *Eur. Child. Adolesc. Psychiatry* 22 567–575. 10.1007/s00787-013-0400-3 23532400

[B81] Ying L. Wu X. Lin C. Jiang L. (2014). Traumatic severity and trait resilience as predictors of posttraumatic stress disorder and depressive symptoms among adolescent survivors of the Wenchuan earthquake. *PLoS ONE* 9:e89401 10.1371/journal.pone.0089401 PMC3935868 24586751

[B82] Yu X. Zhang J. (2007). Factor analysis and psychometric evaluation of the Connor-Davidson resilience scale (CD-RISC) with Chinese people. *Soc. Behav. Pers.* 35 19–30. 10.2224/sbp.2007.35.1.19

[B83] Zhou Y. Han Q. Fan F. (2016). Latent growth curves and predictors of depressive symptoms among Chinese adolescent earthquake survivors. *Pers. Individ. Dif.* 100 173–178. 10.1016/j.paid.2016.02.009 PMC7114640 32287652

